# Mechanical and Mineralogical Properties of Concrete Incorporating Low-Lime Calcium Silicate Cement Under Accelerated Carbonation Curing

**DOI:** 10.3390/ma19194239

**Published:** 2026-10-06

**Authors:** Sang-Rak Sim

**Affiliations:** Department of Architectural Engineering, Daejin University, Pocheon-si 11159, Gyeonggi-do, Republic of Korea; simsr@daejin.ac.kr; Tel.: +82-31-539-1940

**Keywords:** low-lime calcium silicate cement (CSC), accelerated carbonation curing, mechanical properties, CO_2_ uptake

## Abstract

Low-lime calcium silicate cement (CSC) offers the potential to reduce CO_2_ emissions during cement production and sequester CO_2_ through carbonation curing. However, the relationship between CSC replacement, strength development, and CO_2_ uptake requires clarification at the concrete scale. This study evaluated the effects of replacing OPC with 0%, 25%, and 50% CSC on compressive strength, carbonation behavior, phase evolution, and CO_2_ sequestration in concrete. Following initial steam curing, specimens underwent carbonation curing under 20% CO_2_ for up to 14 days. Compressive strength and carbonation depth were measured, while phase evolution and CO_2_ uptake were examined using X-ray diffraction, Fourier-transform infrared spectroscopy, and thermogravimetric analysis (TGA). After 14 days, compressive strengths were 35.46, 29.84, and 27.95 MPa, respectively. Although final strength decreased with increasing CSC replacement, the 50% mixture exhibited an approximately 398% increase from its pre-carbonation strength of 5.61 MPa. Corresponding carbonation depths were 8.8, 18.2, and 27.1 mm, and TGA-based net CO_2_ uptakes were 18.2, 34.0, and 50.5 kg CO_2_/t-concrete. Calcite and vaterite were identified, while infrared spectral changes suggested the formation of silica-rich reaction products. Thus, 50% OPC replacement achieved approximately 28 MPa and 2.8 times the CO_2_ uptake of the control under the investigated conditions. These findings demonstrate the potential to combine OPC substitution with mineral CO_2_ sequestration, while highlighting a trade-off between compressive strength and CO_2_ uptake. Further mechanical and durability evaluations and a comprehensive life-cycle assessment are required to establish structural applicability and net greenhouse gas emission reductions.

## 1. Introduction

Concrete is the most widely used construction material worldwide, and its demand is expected to continue increasing with industrial development, urbanization, and population growth [1,2,3,4,5]. Cement, the primary binder in concrete, is produced by calcining a mixture of limestone (CaCO_3_) and clay-based raw materials containing SiO_2_, Al_2_O_3_, Fe_2_O_3_, and other constituents at 1450 °C, with its production emitting approximately 700–800 kg of CO_2_ per ton of clinker [6]. In terms of CO_2_ emissions associated with cement production, approximately 60% result from the thermal decomposition of limestone, while approximately 33% are attributed to fossil-fuel combustion during clinker production [7,8,9]. Together, these sources account for the majority of cement-related CO_2_ emissions. Moreover, the cement industry is responsible for approximately 8% of global CO_2_ emissions and is therefore recognized as one of the most carbon-intensive industrial sectors [10].

In response to global efforts to achieve carbon neutrality by 2050, the cement industry is actively pursuing various strategies to reduce its greenhouse gas emissions. Among these strategies, mineral carbonation-based carbon capture utilization and sequestration (CCUS) has attracted considerable attention because it chemically binds CO_2_ in the form of thermodynamically stable carbonate minerals [11].

In conventional reinforced concrete structures, carbonation is generally regarded as a deterioration mechanism because it reduces the alkalinity of the cover concrete, destabilizes the passive film surrounding the reinforcing steel, and may consequently initiate reinforcement corrosion. However, under appropriately controlled early-age curing conditions, carbonation can have beneficial effects on cementitious materials. CaCO_3_ formed through the reaction of calcium-bearing phases with CO_2_ can fill pores and densify the microstructure, potentially increasing strength and reducing permeability [12,13,14,15,16].

To reduce CO_2_ emissions associated with both cement production and subsequent curing, CO_2_-reactive low-lime calcium silicate cement (CSC) has recently been proposed as a promising low-carbon binder, particularly for precast concrete applications. The calcination temperature of CSC is approximately 1250 °C, which is approximately 200 °C lower than that of ordinary Portland cement (OPC), thereby reducing the energy demand and fuel-related CO_2_ emissions associated with clinker production. In addition, CSC requires approximately 30% less limestone than OPC, resulting in a further reduction in process-related CO_2_ emissions. The primary mineral phases of CSC are rankinite (C_3_S_2_) and wollastonite (CS), both of which exhibit limited hydraulic reactivity. Unlike OPC, which primarily develops strength through hydration, CSC develops its mechanical properties mainly through carbonation curing. During this process, its calcium silicate phases react with CO_2_ in the presence of moisture to form CaCO_3_ and an amorphous silica-rich phase [17,18].

Because the strength development of CSC depends predominantly on carbonation, an appropriate carbonation-curing system is essential for achieving the required mechanical performance. CSC is therefore considered particularly suitable for precast concrete products, for which curing conditions can be precisely controlled. Conventional OPC-based precast products generally achieve rapid strength development through accelerated hydration under steam-curing conditions. In contrast, CSC-based products require rapid and relatively uniform CO_2_ penetration and carbonation to ensure sufficient early-age strength and production efficiency. Accordingly, carbonation environments with CO_2_ concentrations of 20% or higher have frequently been employed to accelerate the carbonation of CSC-based materials. Increasing the CO_2_ concentration increases the concentration gradient between the curing environment and the pore system, thereby potentially promoting CO_2_ ingress, the formation of carbonation ions in the pore solution, and the subsequent carbonation of calcium silicate phases [19].

Previous studies have investigated the carbonation behavior, reaction products, and strength development of CO_2_-reactive non-hydraulic cement systems. Ashraf et al. [20] examined CSC pastes and mortars and identified calcium carbonate and Ca-modified silica gel as the principal carbonation products. They evaluated the effects of water-to-cement ratio, temperature, relative humidity, and CO_2_ concentration on carbonation kinetics and reported mortar compressive strengths of up to approximately 40 MPa after three days of carbonation curing. Their findings demonstrated the potential of CSC to develop strength through carbonation and highlighted the importance of curing conditions.

Regarding blended cement systems, Cho et al. [18] reported that CSC paste incorporating 20 wt.% OPC exhibited the highest compressive strength after carbonation curing, reaching up to 2.5 times that of pure CSC. They attributed this improvement to strong mechanical interlocking between rhombic CaCO_3_ crystals, further reinforced by vaterite. Kim et al. [21] investigated the reaction and microstructural characteristics of OPC–CSC pastes under 5% CO_2_ curing. Their results indicated that OPC dissolution governed the initial reaction, whereas CSC reaction became evident between one and three days of curing. Pastes containing at least 30 wt.% CSC generally exhibited increasing compressive strength and decreasing total porosity with continued carbonation curing. More recently, Adem et al. [22] examined OPC pastes containing 0–50 wt.% CSC under 20% CO_2_ curing. After seven days, the paste containing 50 wt.% CSC achieved a compressive strength of 45.6 MPa, compared with 26.0 MPa for the OPC reference, accompanied by increased carbonate formation and microstructural refinement.

Focusing on carbonation-curing conditions, Kim et al. [23] conducted a preliminary study aimed at identifying optimal curing conditions by evaluating the effects of CO_2_ concentration, a key factor governing carbonation. Following steam curing, CSC-containing cement pastes were subjected to carbonation curing at CO_2_ concentrations of 20% and 99% for up to 24 h, and their compressive strength, carbonation depth, and CO_2_ uptake were evaluated.

Taken together, these studies suggest that strength development in OPC–CSC systems depends on the sequence and balance of hydration and carbonation reactions. During carbonation curing, CO_2_ dissolves in pore water and lowers the local pH, facilitating the dissolution of reactive calcium silicate phases in CSC and the release of Ca^2+^. The released calcium reacts with dissolved carbonate species to precipitate CaCO_3_, while calcium removal and reorganization of the silicate structure lead to the formation of silica-rich phases, including Ca-modified silica gel. In blended systems, OPC hydration produces an initial binding matrix containing C–S–H and Ca(OH)_2_. Subsequent carbonation can consume Ca(OH)_2_ and induce decalcification of C–S–H, providing additional calcium for carbonate precipitation and modifying the existing hydration products. Carbonate precipitation within the hydrated matrix and at particle contacts can contribute to pore filling and mechanical interlocking, whereas extensive decalcification of C–S–H may adversely affect the binding matrix. Using CSC as the sole binder offers the potential to reduce CO_2_ emissions associated with cement production and to sequester CO_2_ through carbonation curing. However, in the wet-cast concrete production process adopted in this study, sufficient early strength is required to enable demolding before carbonation curing, allowing the concrete surfaces to be exposed to CO_2_. Incorporating OPC promotes early strength development through hydration during initial steam curing, thereby enabling demolding followed by further strength development and CO_2_ fixation through carbonation curing.

Despite these advances, findings from paste and mortar systems and dedicated CSC concrete products do not directly establish the performance of concrete in which OPC is partially replaced with CSC and steam curing precedes carbonation curing. Increasing CSC replacement reduces the proportion of OPC available for initial hydration while increasing the proportion of calcium silicate phases that depend primarily on carbonation for strength development. Consequently, the balance between initial strength, subsequent strength development, and CO_2_ uptake requires direct evaluation under the selected concrete mixing and curing conditions. It is necessary to determine whether the increased extent of carbonation and CO_2_ uptake associated with CSC replacement is accompanied by comparable gains in compressive strength.

Accordingly, this study investigated concrete with OPC replacement levels of 0%, 25%, and 50% CSC subjected to initial steam curing followed by carbonation curing at a CO_2_ concentration of 20% for up to 14 days. Compressive strength and carbonation depth were evaluated, carbonation-related changes in phase composition and silicate structure were examined using XRD and FT-IR, and net CO_2_ uptake was quantified using TGA. The contribution of this study is to clarify the relationship between strength development and CO_2_ sequestration as CSC replacement increases in OPC–CSC blended concrete. By relating these performance measures to the observed carbonation products, the study provides material-level evidence to inform the selection of CSC replacement levels and the further development of carbonation-curing strategies for precast applications.

## 2. Experimental Program

### 2.1. Materials and Preparation

The binder materials used in this study were Type I OPC (HANIL Cement, Seoul, Republic of Korea) and CSC. The CSC was experimental cement manufactured using limestone and silica fume as the principal calcium and silica sources, respectively. The prepared raw meal was calcined at 1250 °C in a pilot-scale kiln operated by the Korea Institute of Limestone & Advanced Materials. The chemical compositions of the OPC and CSC, determined using X-ray fluorescence spectroscopy (XRF, Simultix 14, Rigaku, Tokyo, Japan), are presented in Table 1. The OPC contained 61.2 wt.% CaO and 20.7 wt.% SiO_2_, whereas the CSC contained 41.5 wt.% CaO and 53.2 wt.% SiO_2_. Thus, compared with OPC, CSC was characterized by a lower CaO content and a substantially higher SiO_2_ content, consistent with its low-lime calcium silicate composition.

**Figure 1:**
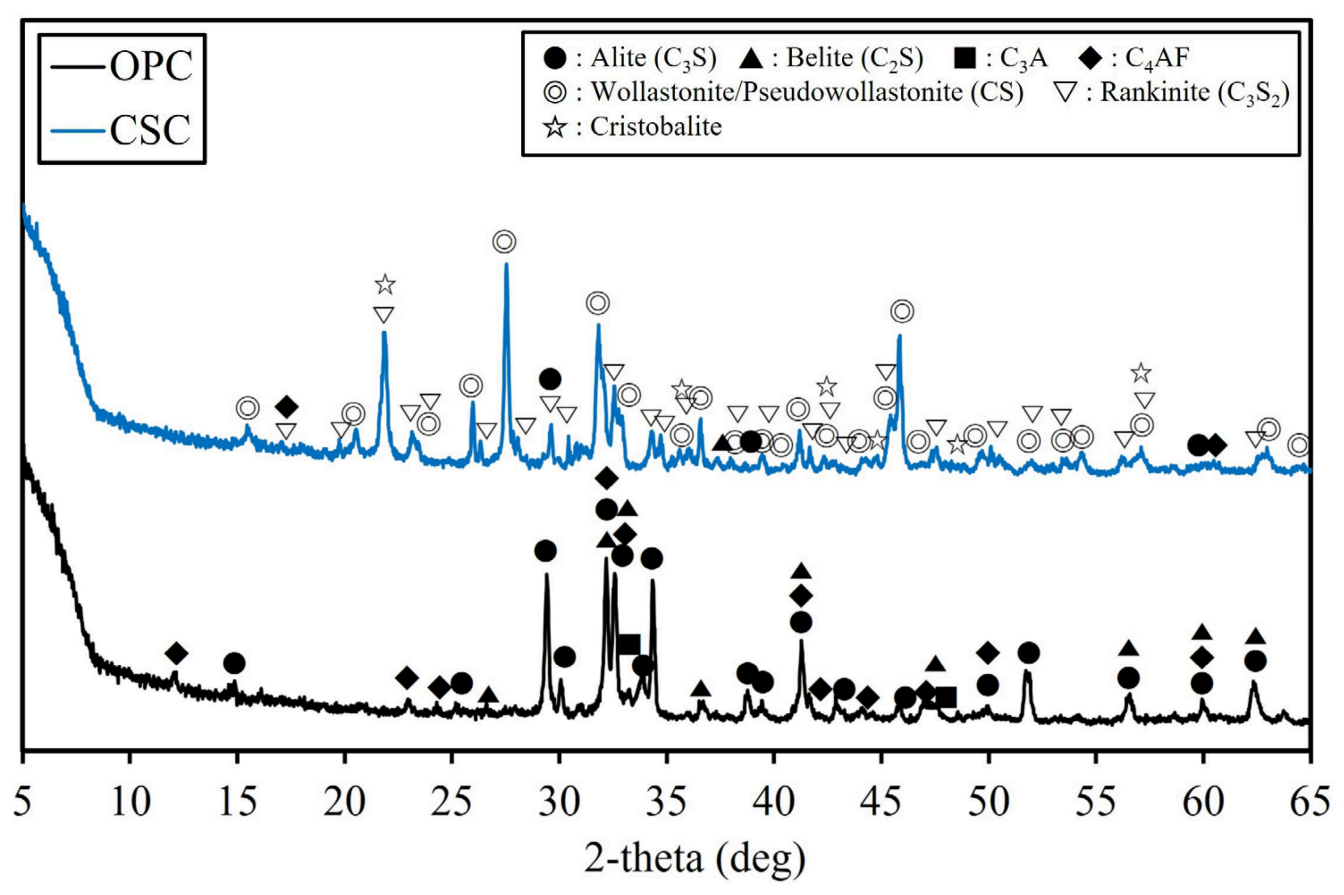
XRD patterns of OPC and CSC.

Figure 1 presents the X-ray diffraction (XRD) patterns of OPC and CSC. The OPC was characterized by peaks attributed to hydraulic clinker phases, including Alite (C_3_S), Belite (β-C_2_S), Tricalcium aluminate (C_3_A) and Brownmillerite (C_4_AF). In contrast, the CSC exhibited peaks corresponding mainly to non-hydraulic calcium silicates, including Pseudowollastonite (CS) and Rankinite (C_3_S_2_).

**Figure 2:**
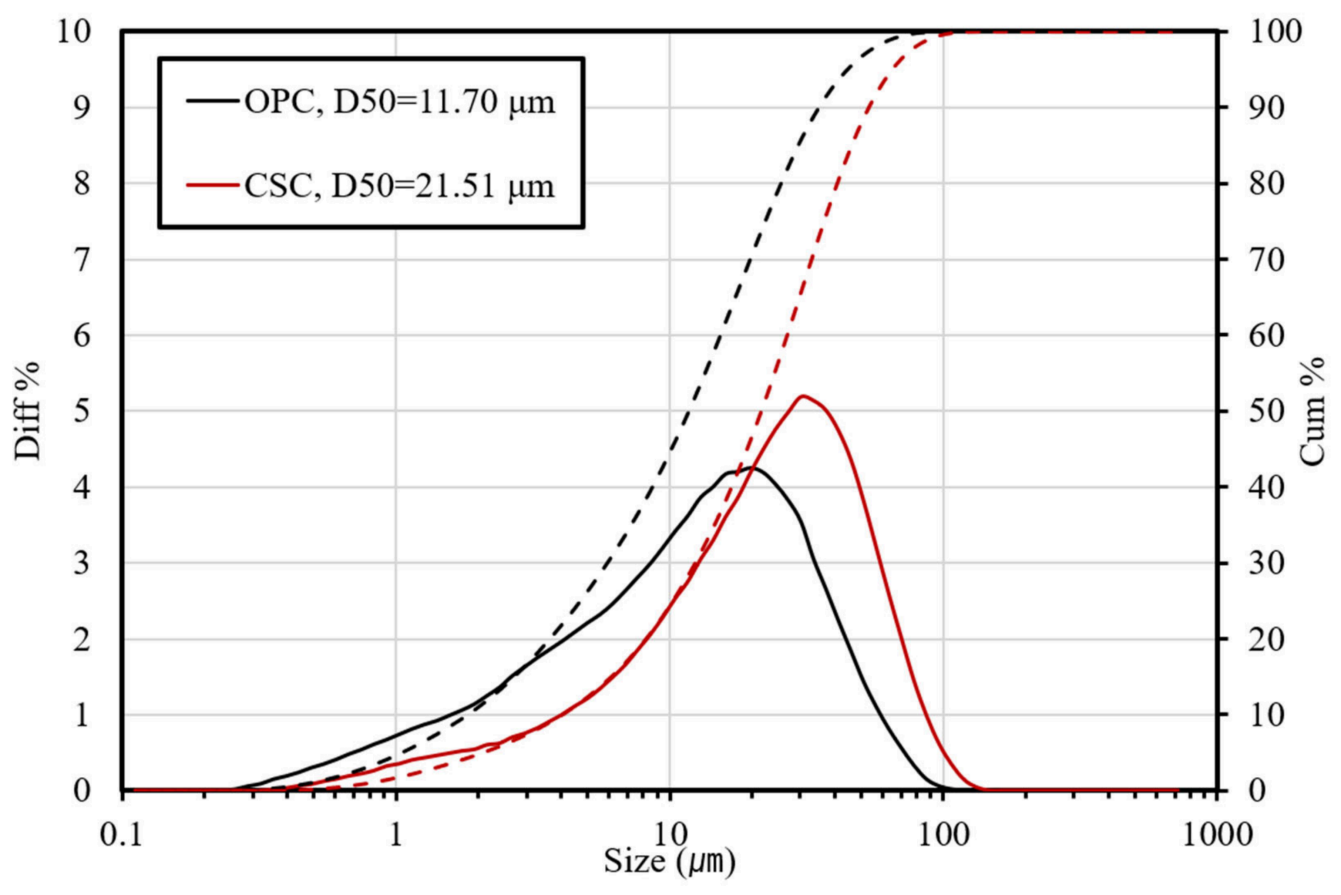
Particle size distribution of OPC and CSC powders (solid lines: particle size distributions; dashed lines: cumulative distributions).

The particle size distributions of OPC and CSC are presented in Figure 2. The D_50_ values of OPC and CSC were 11.70 and 21.51 μm, respectively, indicating that CSC had a larger median particle size than OPC.

Natural river sand (S) and natural crushed stone (G) were used as the fine and coarse aggregates, respectively. The apparent densities of S and G were 2.61 and 2.59 g/cm^3^, respectively, while their fineness moduli were 2.95 and 7.02. The physical properties of the aggregates are summarized in Table 2.

### 2.2. Sample Preparation

This study investigated the effects of partially replacing OPC with CSC on the mechanical properties of concrete subjected to accelerated carbonation curing. The CSC replacement ratios were set at 0%, 25%, and 50% by mass of the total binder, and the mixtures were designated as CSC0, CSC25, and CSC50, respectively. The water-to-binder ratio (W/B), fine aggregate ratio (S/a), unit water content, and total binder content were fixed at 45.5%, 48.6%, 167 kg/m^3^, and 367 kg/m^3^, respectively. A liquid polycarboxylate acid-based superplasticizer (SP) and a high-range AE water-reducing agent (AE) were added at dosages adjusted to achieve a target slump of 180 ± 25 mm and a target air content of 4.5 ± 1.5%. The mix proportions are presented in Table 3.

For each combination of mixture composition and carbonation-curing age, four cylindrical concrete specimens (Φ100 mm × 200 mm) were prepared. Three specimens were used for compressive strength testing, and the remaining specimen was used to determine the carbonation ratio. For mineralogical analysis, three paste specimens (50 mm × 50 mm × 50 mm) were prepared for each condition to exclude the influence of aggregates on the analysis.

**Figure 3:**
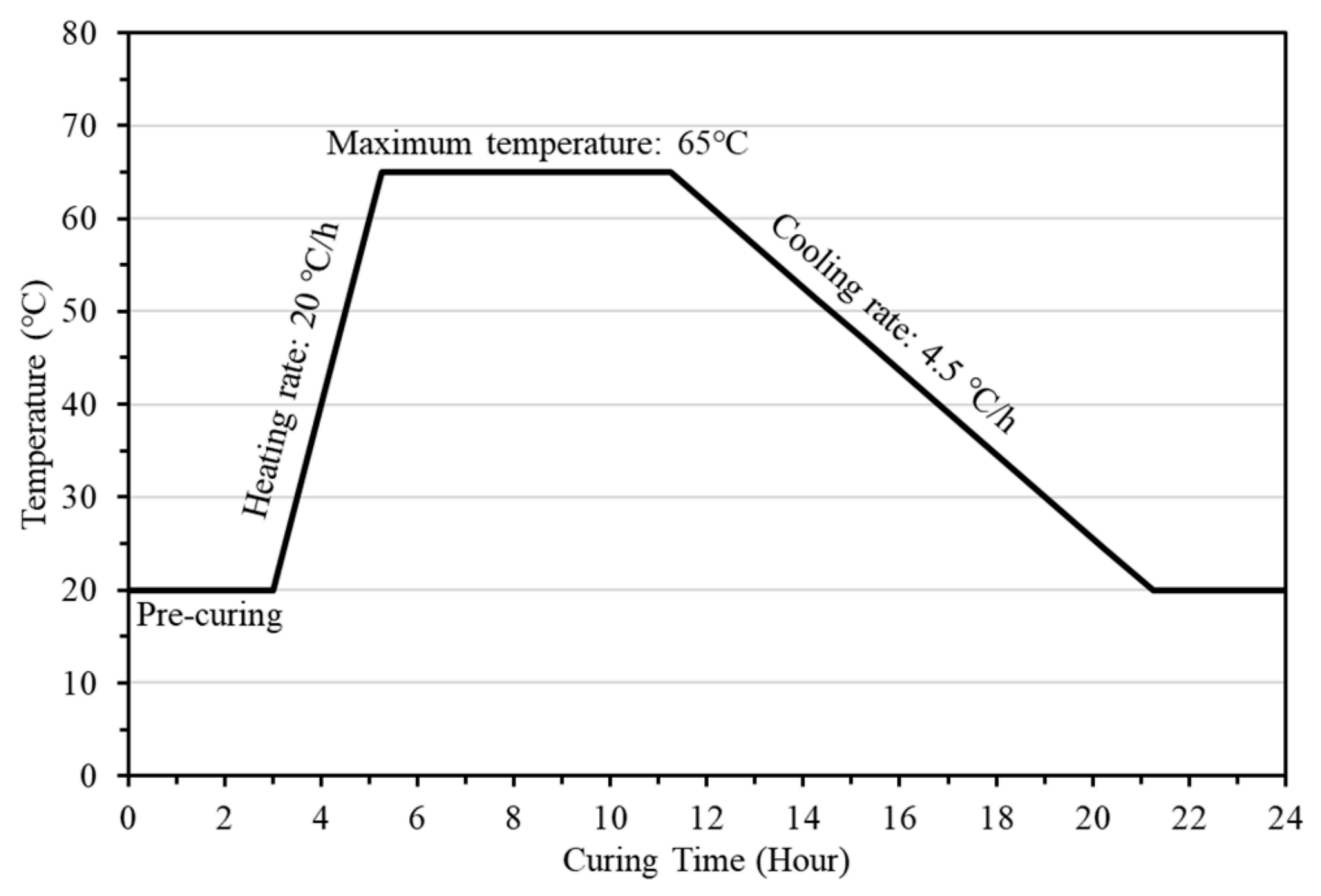
Steam-curing method.

To reflect the manufacturing process of precast concrete products, all specimens were initially steam-cured intact in their molds, without being cut into smaller pieces. As shown in Figure 3, steam curing was conducted for a total of 24 h, with an initial temperature of 20 °C and a maximum temperature of 65 °C. The heating and cooling rates were 20 °C/h and 4.5 °C/h, respectively. This initial steam-curing treatment was intended to accelerate OPC hydration and promote early strength development before carbonation curing. After steam curing, the specimens were demolded and transferred to a carbonation chamber maintained at a CO_2_ concentration of 20%, a temperature of 20 °C, and a relative humidity of 60%. Because maintaining precast products in a carbonation chamber for up to 28 days would reduce production efficiency and limit their practical applicability, the maximum carbonation-curing period was set to 14 days. Accordingly, carbonation curing was conducted for 1, 3, 7, and 14 days.

### 2.3. Test Methods

#### 2.3.1. Evaluation of Carbonation Depth

**Figure 4:**
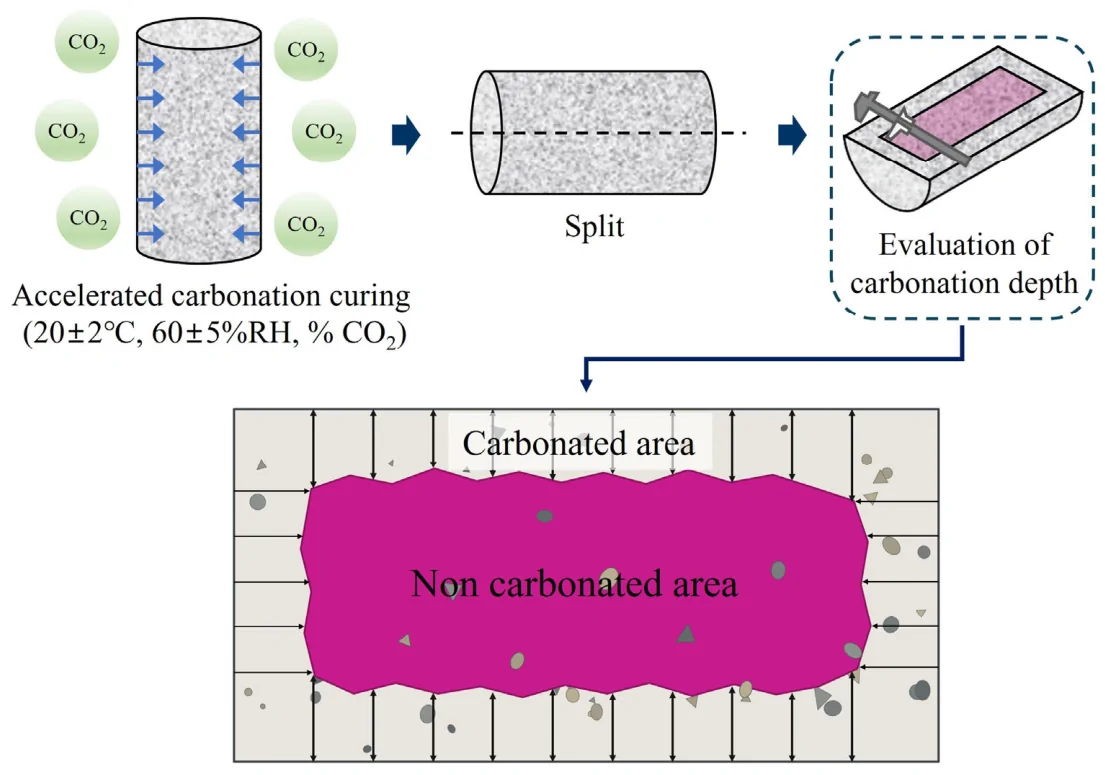
Measurement of the carbonation depth.

To measure the carbonation depth, the specimens (Φ100 mm × 200 mm) were split longitudinally at each designated accelerated carbonation-curing age, and a 1% phenolphthalein solution was sprayed onto the freshly exposed surfaces. After color development, the carbonation depth was determined as the perpendicular distance from the outer surface to the boundary between the carbonated and non-carbonated regions. Measurements were taken at approximately 10 mm intervals along the four edges of the longitudinally split surface, at locations where the perpendicular measurement lines intersected the boundary of the remaining non-carbonated region. The number of measurement locations varied with the size and geometry of this region and decreased as carbonation progressed. A total of 12–30 measurements were obtained per split surface, and their arithmetic mean was reported as the carbonation depth. Figure 4 schematically illustrates the measurement procedure.

#### 2.3.2. Mechanical Properties

The compressive strength test of the concrete specimens (Φ100 mm × 200 mm) was measured at each designated carbonation-curing age using a universal testing machine at a loading rate of 0.6 MPa/s [24]. Three specimens were tested for each mixture at each age, and the mean value was reported as the compressive strength.

#### 2.3.3. Mineralogical Properties

##### X-Ray Diffraction (XRD)

To evaluate changes in mineralogical phase composition induced by accelerated carbonation curing, X-ray diffraction (XRD) analysis was conducted on paste specimens prepared with the same W/B ratio and binder proportions as the corresponding concrete mixtures. Paste specimens were used to minimize interference from aggregate-derived diffraction peaks and facilitate the identification of carbonation reaction products. Samples for XRD analysis were collected immediately after steam curing (Ctrl, before carbonation curing) and after 3 and 14 days of accelerated carbonation curing. XRD measurements were performed using an X-ray diffractometer (D/max 2002, Rigaku, Japan) with Cu Kα radiation (λ = 1.5410 Å) operated at 40 kV and 30 mA. The diffraction patterns were recorded over a 2θ range of 10–65°, with a step size of 0.02° and a counting time of 1.5 s per step.

Rietveld refinement was performed using HighScore Plus software (version 5.3) for quantitative phase analysis. ZnO was mixed with each sample as an internal standard at 20 wt.% of the total powder mixture, and the amorphous content was estimated using the internal standard method. The agreement between the measured and calculated diffraction patterns was assessed using the goodness of fit (GOF), with a GOF of 2.0 or lower adopted as the profile-fitting criterion.

##### Fourier-Transform Infrared (FT-IR) Spectroscopy

FT-IR spectroscopy was performed to evaluate changes in chemical bonding environments and reaction products induced by accelerated carbonation curing. The analysis was conducted using an FT-IR spectrometer (Nicolet iS50, Thermo Scientific, Madison, WI, USA) over a wavenumber range of 400–4000 cm^−1^. The same paste samples prepared for XRD analysis were used for the FT-IR measurements. These samples were collected immediately after steam curing (Ctrl, before carbonation curing) and after 3 and 14 days of accelerated carbonation curing.

##### Thermogravimetric Analysis (TGA)

TGA was conducted to quantify the amount of CO_2_ fixed in the concrete through accelerated carbonation curing. After 14 days of accelerated carbonation curing, all fragments from the three concrete specimens subjected to compressive strength testing using a universal testing machine were collected for each mixture and processed together. The fragments were coarsely crushed using a jaw crusher and further pulverized in a jet mill to obtain particles smaller than 75 μm. The resulting powder was mixed and homogenized to prepare a single composite powder sample for each mixture. A single TGA measurement was performed on an aliquot of each composite powder sample. The resulting powder was homogenized in a ball mill for 15 min, after which a representative portion was collected for analysis. TGA was performed using a thermogravimetric analyzer (STA 2500, NETZSCH, Selb, Germany) over a temperature range of 25–1000 °C at a heating rate of 10 °C/min. The amount of fixed CO_2_ was determined from the mass loss associated with the thermal decomposition of carbonate phases.

## 3. Results

### 3.1. Carbonation Depth

**Figure 5:**
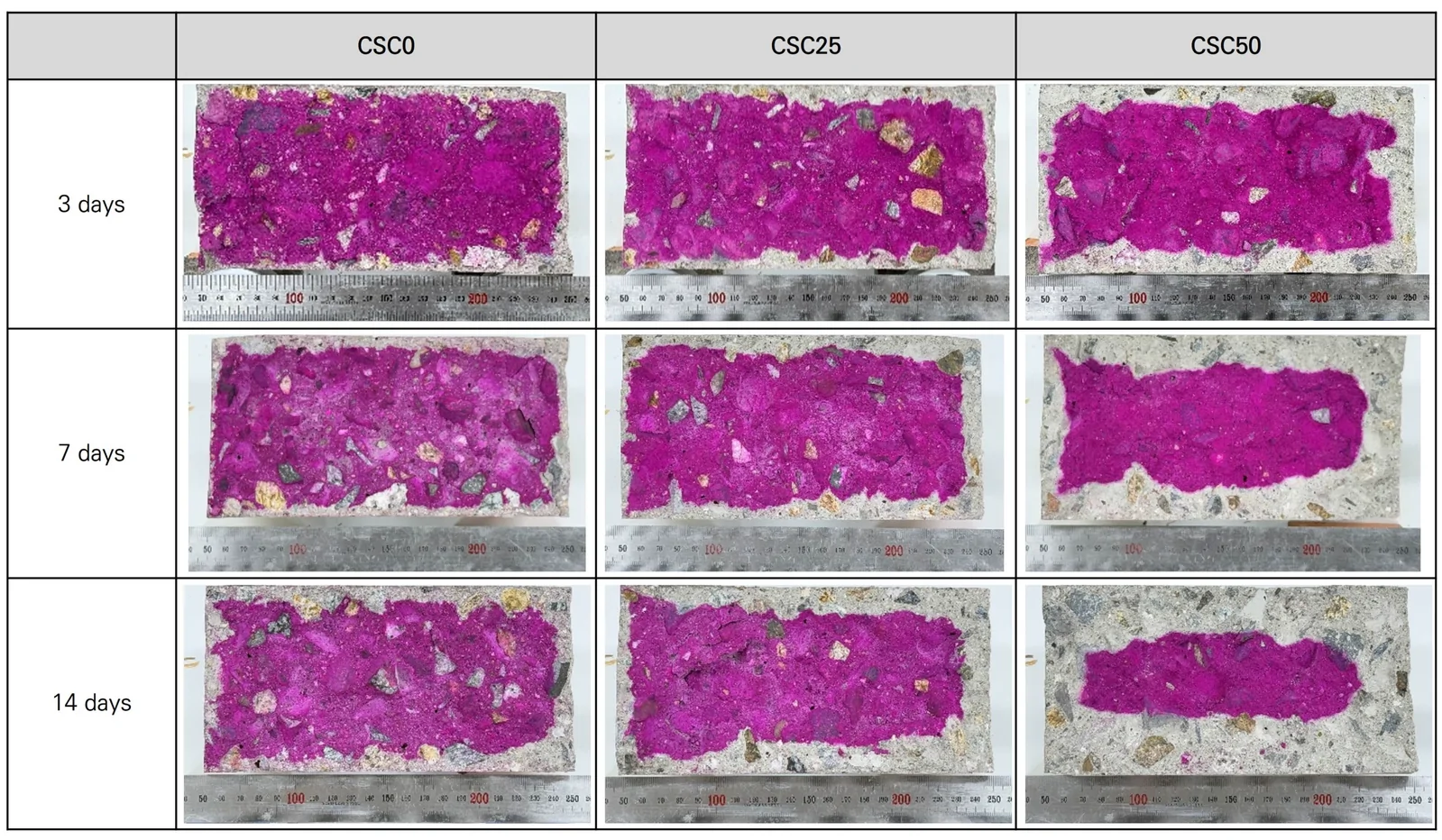
Phenolphthalein-stained cross sections of concrete specimens after accelerated carbonation curing.

**Figure 6:**
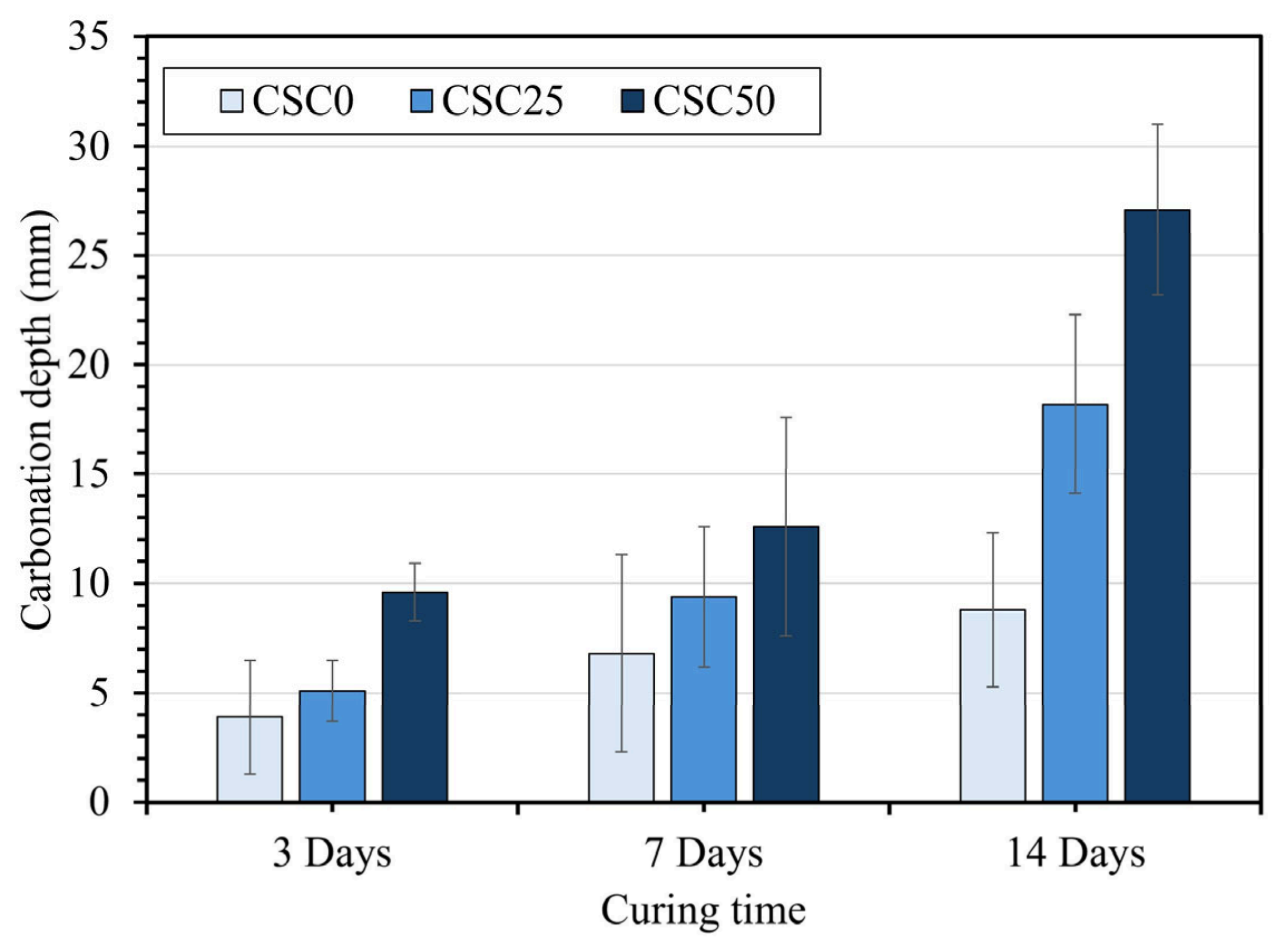
Carbonation depths of concrete specimens after accelerated carbonation curing.

The carbonation depths of concrete specimens at different accelerated carbonation-curing ages are shown in Figure 5 and Figure 6. After 3 days of carbonation curing, the carbonation depths of CSC0, CSC25, and CSC50 were 3.9, 5.1, and 9.4 mm, respectively. These values increased to 6.8, 9.4, and 12.6 mm after 7 days and to 8.8, 18.2, and 27.1 mm after 14 days, respectively. The carbonation depth increased with the carbonation-curing period for all mixtures. At the same curing age, the carbonation depth also tended to increase with increasing CSC replacement ratio.

In CSC0, which contained OPC as the sole binder, steam curing accelerated OPC hydration and promoted the formation of hydration products, including C–S–H, resulting in a relatively dense microstructure. This microstructural densification restricted CO_2_ diffusion pathways and consequently resulted in a lower carbonation depth in CSC0 than in the CSC-containing mixtures.

In contrast, the partial replacement of OPC with CSC reduced the content of the hydraulic binder in CSC25 and CSC50. Because non-hydraulic CSC exhibits limited formation of reaction products through conventional hydration under steam-curing conditions, the CSC-containing mixtures may have developed a more porous and interconnected pore structure than CSC0 immediately after steam curing. Cho et al. [18] and Kim et al. [21] investigated the mechanical properties and microstructural evolution of OPC–CSC pastes. Their findings suggest that OPC hydration contributes to early matrix densification, whereas increasing the proportion of non-hydraulic CSC may result in a more porous matrix before substantial carbonation occurs. With continued carbonation curing, the formation of carbonation products through reactions involving OPC hydration products and CSC can contribute to matrix densification and a reduction in total porosity.

Similarly, in the present study, CSC25 and CSC50 may have undergone less extensive matrix densification during initial steam curing than CSC0 because of their lower OPC contents. The resulting differences in the initial pore structure could have facilitated CO_2_ ingress and contributed to the greater carbonation depths observed in the CSC-containing mixtures. After 14 days of carbonation curing, the carbonation depths of CSC25 and CSC50 were approximately 106.8% and 208.0% greater than that of CSC0, respectively, indicating that differences in the initial microstructure caused by CSC replacement substantially affected CO_2_ penetration behavior.

The phenolphthalein test provided an indirect indication of carbonation progression in the OPC–CSC mixtures by visualizing regions of reduced alkalinity. However, the measured carbonation depth reflects a decrease in pore-solution pH and does not directly represent the full extent of CO_2_ reaction. In these mixtures, Ca(OH)_2_ formed through OPC hydration contributes to alkaline buffering, whereas the principal CSC phases harden mainly through carbonation. Consequently, differences in the measured depths may reflect variations in alkaline buffering capacity associated with CSC replacement, in addition to differences in CO_2_ transport. The method cannot distinguish the contributions of OPC and CSC or independently quantify the degree of CSC carbonation. Accordingly, the measured depths should be interpreted alongside the XRD, FT-IR, and TGA results and should not be regarded as direct measures of CSC reaction extent or CO_2_ uptake.

### 3.2. Compressive Strength

**Figure 7:**
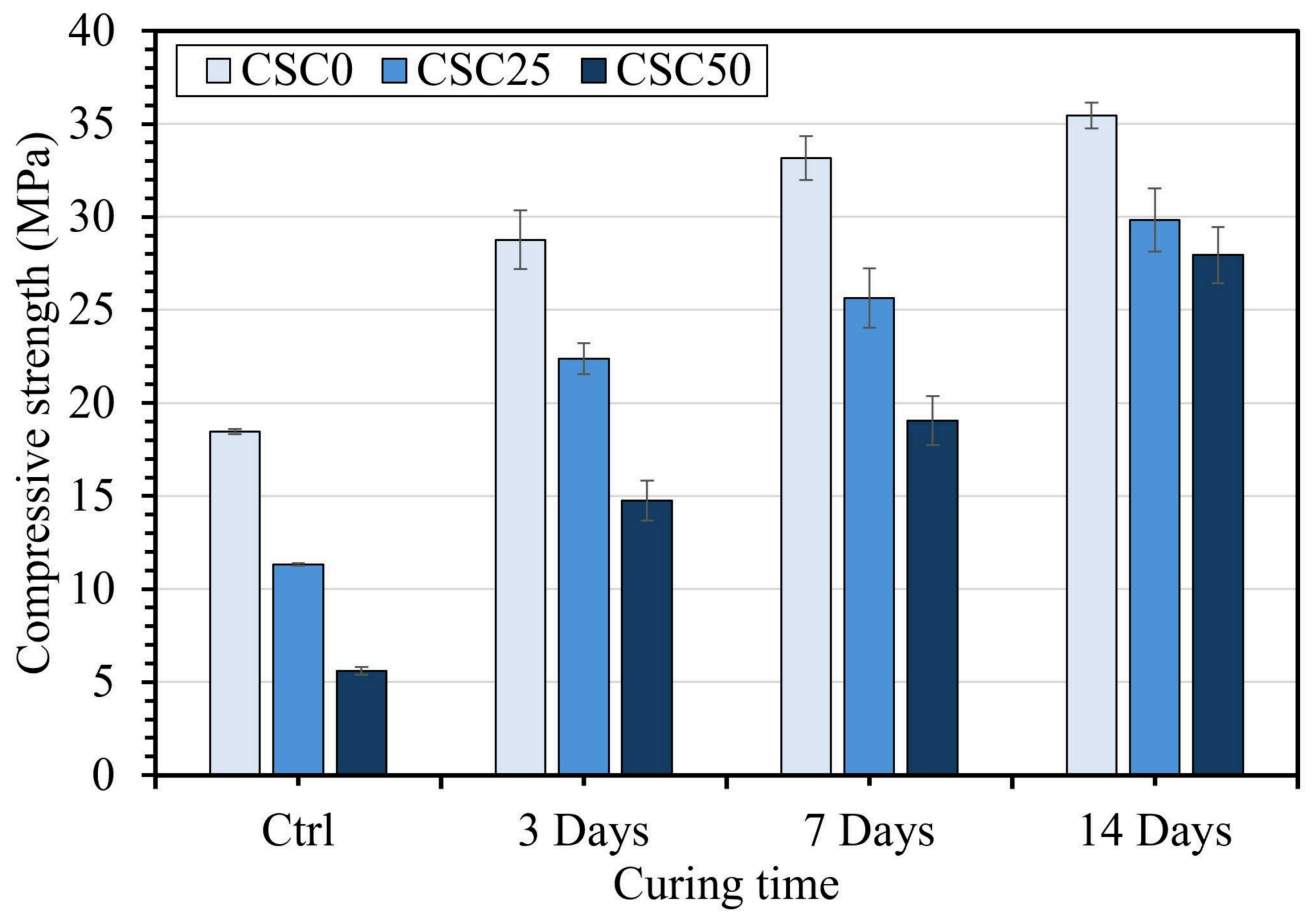
Compressive strength of concrete specimens at 1 day, 3 days, 7 days, and 14 days.

The compressive strengths of the concrete specimens after different periods of accelerated carbonation curing are shown in Figure 7. The results are reported as the mean ± standard deviation of three specimens (*n* = 3), and the error bars in Figure 7 represent ± one standard deviation. Immediately after steam curing and before accelerated carbonation curing (Ctrl), the compressive strengths of CSC0, CSC25, and CSC50 were 18.47 ± 0.15, 11.33 ± 0.07, and 5.61 ± 0.19 MPa, respectively. The initial compressive strength decreased with increasing CSC replacement level. This decrease was attributed to the lower OPC content and the consequent reduction in the formation of hydration products during steam curing, given the limited hydraulic reactivity of CSC.

After 3 days of accelerated carbonation curing, the compressive strengths of CSC0, CSC25, and CSC50 increased to 28.77 ± 1.58, 22.39 ± 0.84, and 14.74 ± 1.07 MPa, respectively. The corresponding strengths after 7 days were 33.17 ± 1.19, 25.63 ± 1.60, and 19.05 ± 1.33 MPa and increased further to 35.46 ± 0.68, 29.84 ± 1.70, and 27.95 ± 1.51 MPa after 14 days. Thus, the compressive strength increased continuously with the carbonation-curing period for all mixtures. Across all mixtures and curing conditions, the standard deviations ranged from 0.07 to 1.70 MPa, with coefficients of variation ranging from 0.62% to 7.26%, indicating relatively limited scatter among the three replicate specimens tested under each condition.

Relative to the values measured immediately after steam curing, the 14-day compressive strengths of CSC0, CSC25, and CSC50 increased by approximately 92.0%, 163.4%, and 398.3%, respectively. However, the particularly large relative increase observed for CSC50 should be interpreted considering its low initial strength. The corresponding 14-day carbonation depths were 8.8, 18.2, and 27.1 mm, respectively. The concurrent increases in carbonation depth and relative strength gain with increasing CSC replacement level suggest that the relatively porous initial microstructure associated with CSC replacement facilitated CO_2_ diffusion into the concrete, allowing the carbonation reaction between CO_2_ and the calcium silicate phases of CSC to occur throughout a larger volume. The resulting CaCO_3_ and silica-rich reaction products filled the internal pores and densified the microstructure, thereby contributing to compressive strength development [25].

### 3.3. Quantitative Phase Analysis After Accelerated Carbonation Curing

**Figure 8:**
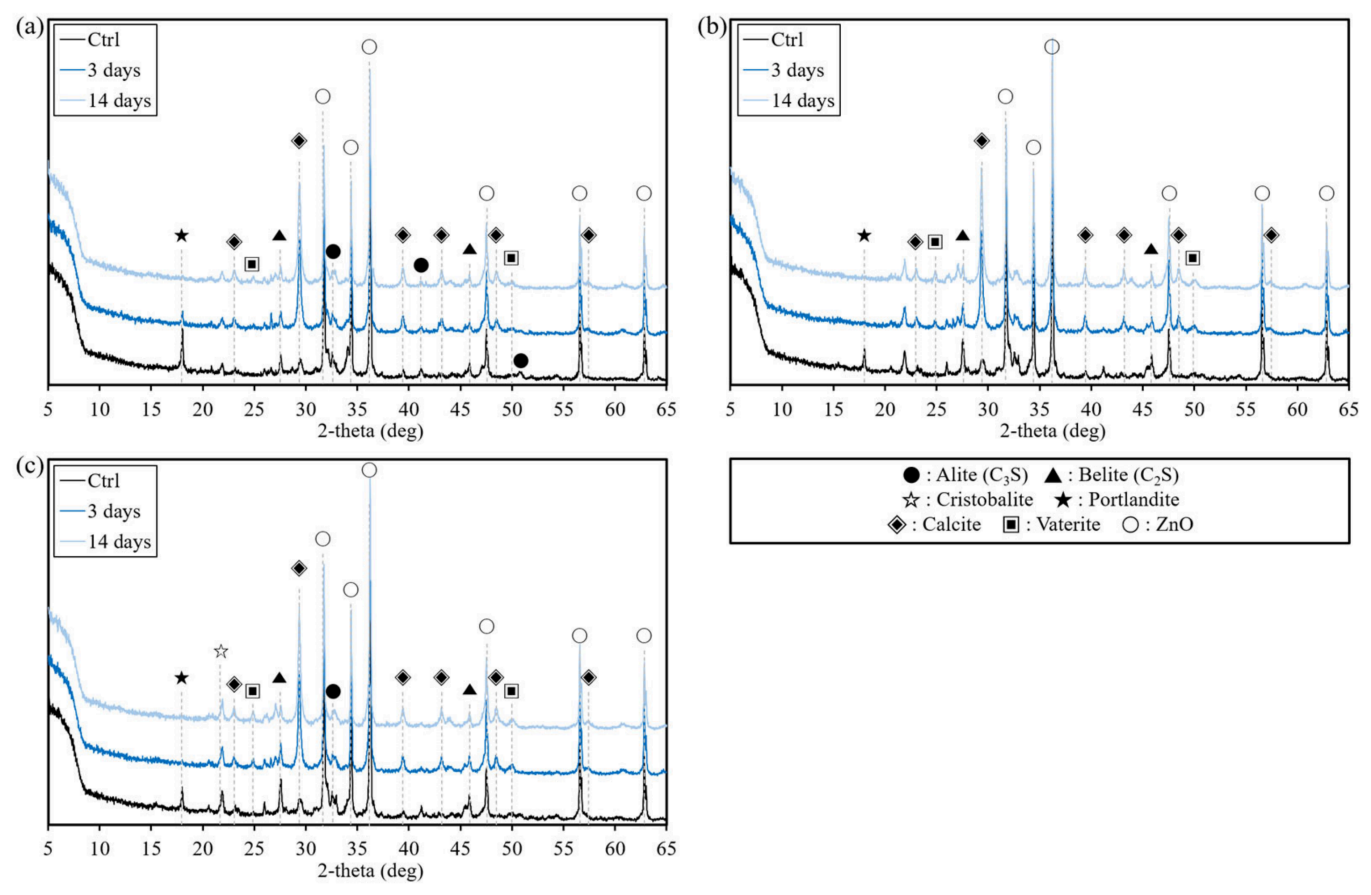
XRD patterns of paste specimens at various accelerated carbonation-curing ages: (**a**) CSC0, (**b**) CSC25, and (**c**) CSC50.

The XRD patterns of the paste specimens immediately after steam curing (Ctrl) and after 3 and 14 days of accelerated carbonation curing are presented in Figure 8. Diffraction peaks corresponding to portlandite, calcite, and vaterite were identified. In addition, zincite (ZnO), which was added as an internal standard for quantitative phase analysis, was detected in all specimens.

For CSC0, a prominent diffraction peak corresponding to portlandite was observed at approximately 18° immediately after steam curing. As the accelerated carbonation-curing period increased, the intensity of the portlandite peak decreased, whereas that of the calcite peak at approximately 29.4° increased markedly. Similar increases in the calcite peak intensity were observed in CSC25 and CSC50 with increasing carbonation-curing period. In addition to calcite, diffraction peaks corresponding to vaterite were identified in the CSC-containing mixtures. A weak vaterite peak was also detected in CSC0 after 14 days of accelerated carbonation curing.

**Figure 9:**
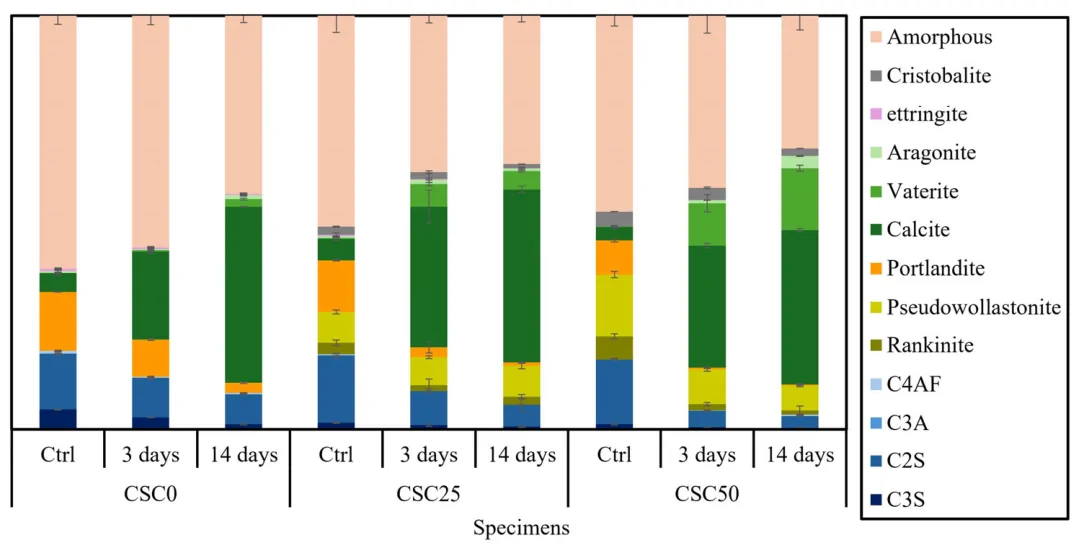
Rietveld-derived quantitative phase compositions of paste specimens at various accelerated carbonation-curing ages.

**Figure A1:**
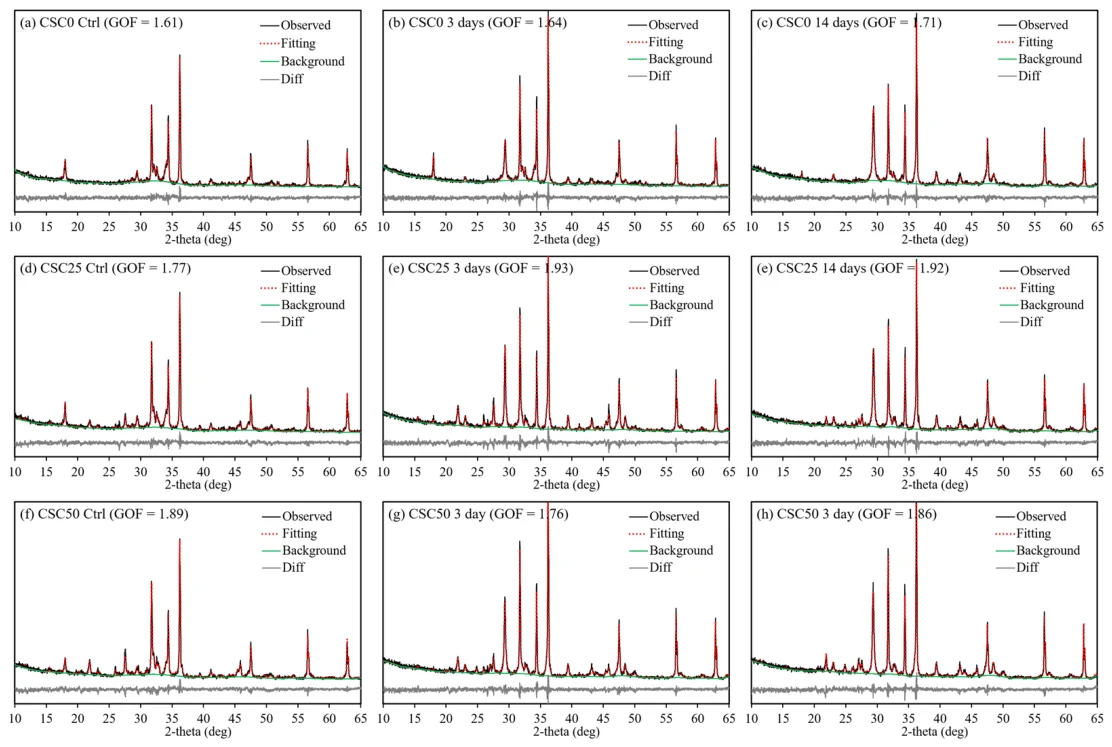
Rietveld refinement results.

The unreacted crystalline phases originally present in CSC and OPC, as well as the crystalline products formed during carbonation curing, were identified from their characteristic diffraction peaks. Rietveld refinement was subsequently performed based on the identified phases, and the resulting quantitative phase compositions are presented in Table 4 and Figure 9. The Rietveld refinement results are presented in Figure A1 (Appendix A).

Immediately after steam curing (Ctrl), amorphous phases and portlandite were identified in all mixtures. This was attributed to the formation of amorphous hydration products, including C–S–H, and portlandite through the accelerated hydration of OPC during steam curing. However, the portlandite content decreased with increasing CSC replacement ratio. This decrease was attributed to the reduced OPC content and the limited contribution of non-hydraulic CSC to conventional hydration under steam-curing conditions. In contrast, the contents of rankinite and pseudowollastonite, the principal clinker phases constituting CSC, increased with increasing CSC replacement ratio.

The most prominent phase changes induced by accelerated carbonation curing were an increase in the calcite content and a decrease in the amorphous phase content. The increase in calcite was attributed to the carbonation of portlandite and C–S–H formed through OPC hydration, as well as the calcium silicate phases of CSC, resulting in the formation of crystalline CaCO_3_. Meanwhile, the decrease in the amorphous phase content may be associated with the decalcification of C–S–H and other amorphous hydration products during carbonation, followed by the conversion of the released Ca into crystalline CaCO_3_ [26,27,28]. Because the carbonation of CSC can also produce silica-rich amorphous phases, the observed decrease suggests that the consumption of the pre-existing amorphous hydration products and their conversion into crystalline carbonates exceeded the formation of new amorphous phases.

After accelerated carbonation curing, the vaterite content also tended to increase with increasing CSC replacement ratio. Calcite was the predominant crystalline carbonation product in CSC0, whereas both calcite and vaterite, a metastable CaCO_3_ polymorph, were identified in CSC25 and CSC50. Previous studies have reported that vaterite or poorly crystalline CaCO_3_ may precipitate during the decalcification of C–S–H [29,30,31]. The preferential precipitation of calcite and vaterite is also influenced by the carbonation environment, including the pH of the reaction system and the local Ca^2+^ concentration [32,33,34]. Higher pH conditions have been reported to favor calcite precipitation, whereas relatively lower pH conditions can favor vaterite formation [32]. The increased vaterite content in the CSC-containing mixtures may therefore be associated with a reduction in the local pH of the hardened matrix. In OPC-rich mixtures, the relatively large amount of Ca(OH)_2_ generated by hydration contributes to maintaining a high pore-solution pH through its buffering action. Conversely, increasing CSC replacement reduces the OPC content and is expected to decrease Ca(OH)_2_ formation and the associated alkaline buffering capacity. This may allow the local pH to decrease more readily during carbonation, creating more favorable conditions for vaterite formation. Accordingly, changes in binder mineralogy and the local reaction environment may have jointly promoted vaterite formation in CSC25 and CSC50. These results indicate that the CSC replacement ratio affected not only the extent of carbonation but also the polymorphic composition of the crystalline CaCO_3_ formed during carbonation curing.

### 3.4. FT-IR Analysis After Accelerated Carbonation Curing

**Figure 10:**
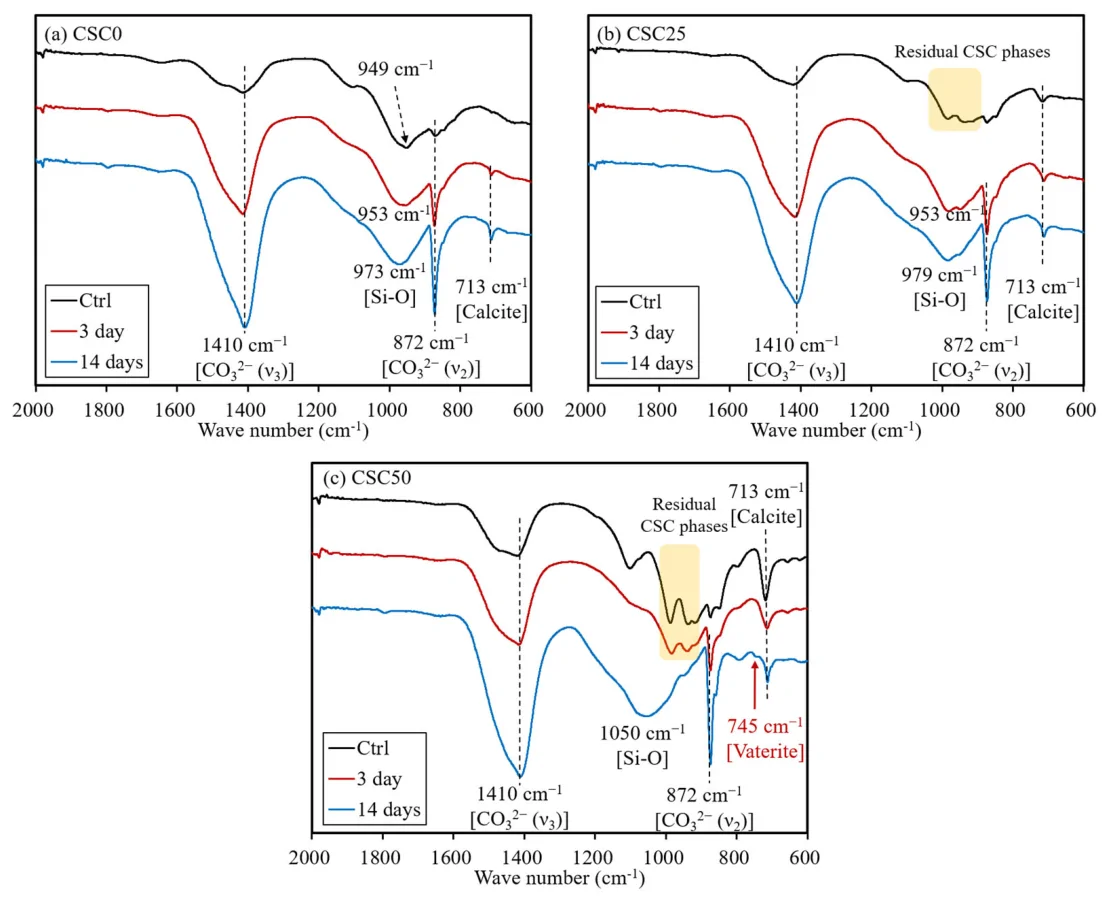
FT-IR spectra of paste specimens: (**a**) CSC0, (**b**) CSC25, and (**c**) CSC50.

The FT-IR spectra of CSC0, CSC25, and CSC50 paste specimens at different accelerated carbonation-curing ages are presented in Figure 10. Immediately after steam curing (Ctrl), CSC0 exhibited a relatively broad Si–O absorption band around 950–1000 cm^−1^, primarily associated with C–S–H formed through OPC hydration. With increasing CSC replacement, several discrete absorption bands became more evident within the Si–O-related region, particularly at approximately 900–1100 cm^−1^. This feature was most pronounced in CSC50 and was attributed to the increasing relative contribution of residual crystalline CSC phases, including rankinite and pseudowollastonite identified by XRD.

Carbonate-related absorption bands were also observed at approximately 1400–1500 and 875 cm^−1^ in all mixtures, corresponding to the asymmetric stretching and out-of-plane bending vibrations of CO_3_^2−^, respectively [35,36]. Compared with Ctrl, these bands became more pronounced after 3 days of accelerated carbonation curing. Further development was observed after 14 days, particularly for the broad band at approximately 1400–1500 cm^−1^. These changes were consistent with continued CaCO_3_ formation over the investigated curing period.

For CSC0, accelerated carbonation curing enhanced the carbonate-related absorption bands, and the characteristic calcite band at approximately 712 cm^−1^ became more evident, consistent with the identification of calcite by XRD. In addition, changes in the shape of the Si–O-related absorption envelope at approximately 950–1100 cm^−1^ may reflect partial decalcification of C–S–H and rearrangement of the silicate structure during carbonation.

For CSC25, changes in the shape and intensity of the Si–O-related absorption envelope at approximately 950–1100 cm^−1^ accompanied the development of the carbonate-related bands. Together with the XRD results, these changes were consistent with the carbonation of both OPC hydration products and the rankinite and pseudowollastonite phases constituting CSC. During carbonation of these calcium silicate phases, Ca released from the silicate structure can precipitate as CaCO_3_, while the residual silicate component can undergo structural rearrangement and polymerization to form a silica-rich gel [20,37].

For CSC50, several of the initially distinct Si–O-related bands became progressively less prominent as accelerated carbonation curing proceeded, whereas the carbonate-related bands intensified markedly. Together with the reduction in residual rankinite and pseudowollastonite contents determined by quantitative XRD, these changes support the consumption of CSC clinker phases through carbonation [20,38,39]. A broad Si–O-related absorption band developed at approximately 1000–1100 cm^−1^ after 14 days of accelerated carbonation curing. The weakening of the discrete bands associated with crystalline calcium silicates, accompanied by the development of this broad band at relatively higher wavenumbers, is consistent with Ca removal from the silicate structure and the formation of a more polymerized silica-rich gel. This spectral change was more evident in CSC50 than in CSC0 and CSC25.

Calcite was identified by XRD in all mixtures, whereas vaterite was additionally identified in CSC25 and CSC50. A characteristic vaterite absorption band was observed at approximately 745 cm^−1^ in CSC50 after 14 days of accelerated carbonation curing. This observation provided additional spectroscopic evidence of vaterite formation and was consistent with the relatively high vaterite content determined for CSC50 by quantitative XRD.

### 3.5. Quantification of CO_2_ Uptake

**Figure 11:**
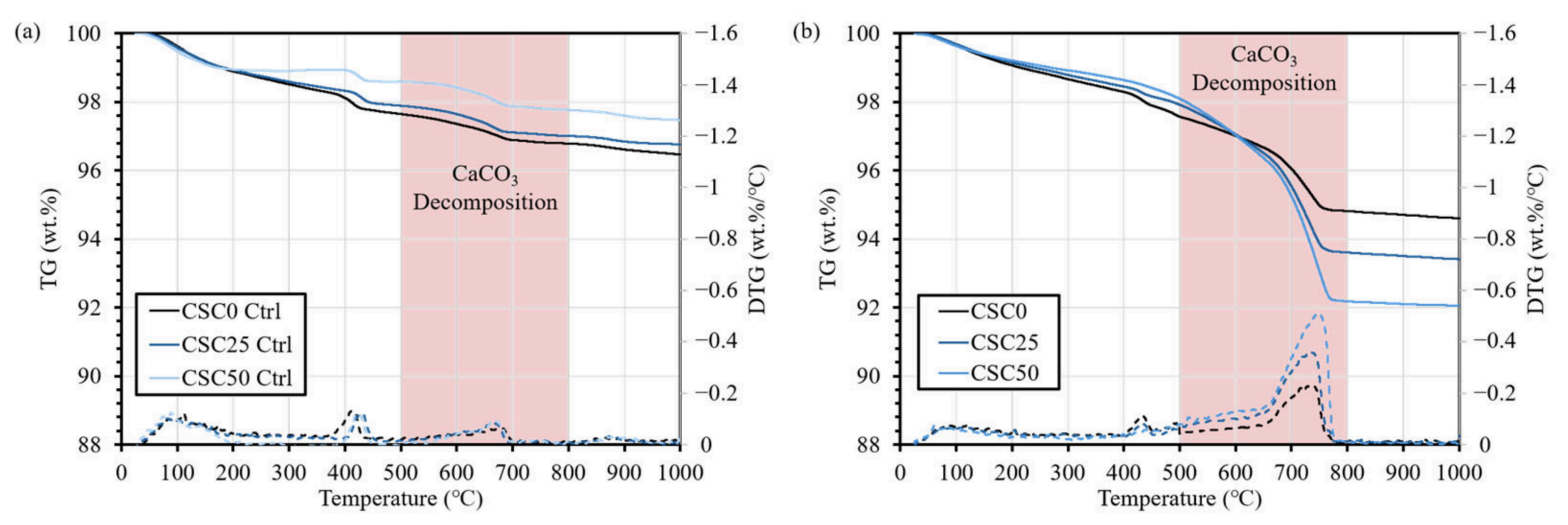
TG and DTG curves of concrete specimens with different CSC replacement ratios: (**a**) before accelerated carbonation curing and (**b**) after 14 days of accelerated carbonation curing.

The TG and DTG results of the concrete specimens before carbonation curing and after 14 days of carbonation curing are presented in Figure 11. Before carbonation curing, all mixtures (CSC0, CSC25, and CSC50) exhibited mass loss in the temperature range of approximately 400–450 °C, primarily attributable to the dehydroxylation of Ca(OH)_2_. After carbonation curing, a distinct mass-loss event within this temperature range was observed only in CSC0, whereas no clearly discernible mass loss attributable to Ca(OH)_2_ dehydroxylation was observed in CSC25 or CSC50. Mass loss attributed to CaCO_3_ decomposition began at temperatures above approximately 500 °C in all mixtures, with a particularly pronounced decrease above approximately 670 °C in the specimens subjected to carbonation curing for 14 days.

Because the thermal decomposition behavior of CaCO_3_ depends on its polymorphic form and crystallinity, the XRD results were considered alongside the TG/DTG profiles when selecting the temperature interval for estimating carbonate-related mass loss. The quantitative phase analysis described above confirmed the presence of vaterite in all mixtures after 14 days of carbonation curing. Considering these observations and the thermal decomposition behavior of calcium carbonates reported previously [20], the mass loss within 500–800 °C was used to estimate the amount of CO_2_ released through carbonate decomposition.

Pre-existing carbonates in concrete may originate from carbonate-bearing aggregates and limestone added as a minor constituent during Portland cement manufacture. KS L 5201 [40] permits limestone additions of up to 5 wt.% of the cement mass. If these pre-existing carbonates are not accounted for, the CO_2_ uptake attributable to carbonation curing may be overestimated. Therefore, specimens of each mixture collected before carbonation curing were used as reference samples to establish the initial carbonate contribution. The mass loss of each reference sample within 500–800 °C was subtracted from that of the corresponding specimen after 14 days of carbonation curing, thereby correcting for the contribution of pre-existing carbonates and estimating the additional CO_2_ fixed during carbonation curing. Both the reference and carbonated specimens underwent the same crushing, grinding, and homogenization procedures before TGA. The mass loss of each reference specimen within 500–800 °C was subtracted from that of the corresponding specimen after 14 days of carbonation curing. The resulting baseline-corrected mass loss was used to estimate the incremental CO_2_ uptake attributable to carbonation curing, as expressed in Equation (1).

(1) $$U_{{CO}_{2}}=[(∆m_{500-800}{)}_{14d}-(∆m_{500-800}{)}_{Ctrl}]\times 10$$

where $U_{CO_{2}}$ is the net CO_2_ uptake resulting from carbonation curing (kg-CO_2_/t-concrete), ${\left(∆m_{500-800}\right)}_{14d}$ is the mass loss within the range of 500–800 °C after 14 days of carbonation curing (wt.%), and ${\left(∆m_{500-800}\right)}_{Ctrl}$ is the mass loss of the corresponding reference specimen within the same temperature range before carbonation curing (wt.%). The conversion factor of 10 in Equation (1) reflects that 1 wt.% of the concrete mass corresponds to 10 kg per ton of concrete.

Before carbonation curing, the mass losses of CSC0, CSC25, and CSC50 within the range of 500–800 °C were 0.87, 0.88, and 0.83 wt.%, respectively, indicating no substantial differences among the mixtures. After 14 days of carbonation curing, the corresponding mass losses increased to 2.69, 4.28, and 5.88 wt.%, respectively. After subtracting the baseline mass losses according to Equation (1), the net mass losses of CSC0, CSC25, and CSC50 were calculated to be 1.82, 3.40, and 5.05 wt.%, respectively. These values correspond to net CO_2_ uptakes of 18.2, 34.0, and 50.5 kg-CO_2_/t-concrete, respectively. The results demonstrate that the net CO_2_ uptake through carbonation curing increased with increasing CSC replacement ratio. The calculated CO_2_ uptake for each mixture is summarized in Table 5.

## 4. Discussion

This study evaluated the compressive strength, carbonation behavior, and CO_2_ uptake of concrete with different CSC replacement levels. Changes in mineralogical phase composition and chemical bonding were investigated using XRD and FT-IR analyses of the corresponding paste specimens. After 14 days of carbonation curing, the compressive strengths of CSC0, CSC25, and CSC50 were 35.46, 29.84, and 27.95 MPa, respectively, indicating a moderate decrease with increasing CSC replacement. This reduction was attributed to the decreased OPC content available for hydration and the limited contribution of non-hydraulic CSC to strength development during the steam-curing stage. Nevertheless, the compressive strength of CSC50 increased by approximately 398%, from 5.61 MPa before carbonation curing to 27.95 MPa after 14 days. These results demonstrate that a compressive strength of approximately 28 MPa can be achieved through carbonation curing even when 50% of the OPC is replaced with CSC, suggesting the potential use of CSC-containing concrete in structural applications. However, its applicability to actual structural members should be further assessed by considering the elastic modulus, tensile and flexural strengths, long-term deformation, and durability in addition to compressive strength.

The mineralogical analyses also confirmed the formation of calcium carbonate and changes in the silicate structure resulting from carbonation curing. Carbonate phases such as calcite and vaterite were detected by XRD, while changes in carbonate and Si–O bonds were observed by FT-IR. These findings suggest that the calcium silicate phases in CSC may have reacted with CO_2_ to form CaCO_3_ and silica-rich reaction products. CaCO_3_ formed through carbonation may precipitate within the pores and contribute to microstructural densification and strength development together with the silica-rich reaction products. However, the current XRD and FT-IR results do not allow the contribution of each reaction product to compressive strength development to be quantitatively distinguished.

After 14 days of carbonation curing, the carbonation depths of CSC0, CSC25, and CSC50 were 8.8, 18.2, and 27.1 mm, respectively, demonstrating that CO_2_ penetration increased with increasing CSC replacement. The net CO_2_ uptakes determined by TGA were 18.2, 34.0, and 50.5 kg-CO_2_/t-concrete, respectively, with CSC50 exhibiting approximately 2.8 times the CO_2_ uptake of CSC0. The reduction in early hydration products and the potentially more open and connected pore structure resulting from CSC replacement likely facilitated CO_2_ penetration into the concrete. Simultaneously, the increased proportion of carbonation-reactive calcium silicate phases in CSC may have contributed to the higher CO_2_ uptake. The consistent increases in carbonation depth, carbonate-phase formation, and TGA-based CO_2_ uptake with increasing CSC replacement support this interpretation.

CSC50 replaced 50% of the OPC with CSC and additionally sequestered 50.5 kg of CO_2_ per ton of concrete through carbonation curing. CSC50 may therefore offer the combined benefits of potentially reducing manufacturing-stage carbon emissions through lower OPC consumption and directly sequestering CO_2_ through mineral carbonation. However, as a preliminary investigation into the practical application of CSC-based concrete, this study focused on material-level performance and quantified only the CO_2_ directly sequestered through carbonation curing using TGA. Greenhouse gas emissions associated with raw-material extraction, CSC calcination, steam curing, CO_2_ separation, compression and transportation, and the energy required for carbonation curing were not included in the assessment. A comprehensive life-cycle assessment covering the entire process from raw-material production to carbonation curing is therefore required to determine the actual carbon-reduction potential of CSC-containing concrete.

In addition, recent studies have explored complementary analytical methods for quantifying CO_2_ fixed in cement-based materials. In the present study, CO_2_ uptake was estimated solely using TGA, without independent quantitative validation using another analytical method. Future investigations should compare TGA-based estimates with acid-digestion-based total inorganic carbon measurements and combustion-based elemental carbon analysis, with appropriate consideration of pre-existing carbonates and, where applicable, organic carbon.

Although this study evaluated the applicability of CSC-containing concrete based on compressive strength, carbonation behavior, mineralogical characteristics, and CO_2_ uptake, its long-term durability, including resistance to chloride penetration, freeze–thaw cycles, and sulfate attack, as well as water absorption and drying shrinkage, was not investigated. Furthermore, the carbonation-curing environment employed a high CO_2_ concentration of 20%. Therefore, the results may not directly represent the long-term CO_2_ penetration, sequestration behavior, and performance of full-scale structural members. Future studies should evaluate members of different dimensions under various carbonation conditions to determine their long-term performance in indoor and outdoor environments and verify their applicability at full scale.

Although carbonation curing can improve concrete strength and CO_2_ uptake, it may also reduce the internal alkalinity of concrete, compromise the passive film on reinforcing steel, and thereby increase the risk of corrosion. Accordingly, before CSC-containing concrete is applied to reinforced concrete structures, depth-dependent pH profiles, reinforcement corrosion potential and corrosion current density, reinforcement–concrete bond behavior, and cracking behavior after carbonation curing should be comprehensively evaluated. Glass-fiber-reinforced polymer (GFRP) bars have recently been proposed as corrosion-resistant alternatives to conventional steel reinforcement. Future studies should therefore investigate the compatibility and applicability of GFRP reinforcement in carbonation-cured CSC-containing concrete.

## 5. Conclusions

This study evaluated the effects of CSC replacement on the compressive strength, carbonation behavior, mineralogical structural characteristics, and CO_2_ sequestration of concrete. The main conclusions are as follows:

- After 14 days of carbonation curing, the compressive strengths of CSC0, CSC25, and CSC50 were 35.46, 29.84, and 27.95 MPa, respectively. Although the final compressive strength decreased with increasing CSC replacement, CSC50 exhibited an approximately 398% increase from its pre-carbonation strength of 5.61 MPa. These results demonstrate that a compressive strength of approximately 28 MPa can be achieved with 50% OPC replacement under the investigated curing conditions.
- XRD identified calcite and vaterite, while FT-IR revealed changes in carbonate and Si–O bonds following carbonation curing. These findings suggest that carbonation of the calcium silicate phases in CSC contributed to the formation of CaCO_3_ and silica-rich reaction products, which may have supported microstructural densification and strength development. However, the individual contributions of these reaction products to compressive strength could not be quantitatively distinguished using the present analyses.
- The carbonation depths of CSC0, CSC25, and CSC50 after 14 days were 8.8, 18.2, and 27.1 mm, respectively, and their corresponding TGA-based net CO_2_ uptakes were 18.2, 34.0, and 50.5 kg-CO_2_/t-concrete. CSC50 exhibited approximately 2.8 times the CO_2_ uptake of CSC0. The concurrent increases in carbonation depth and CO_2_ uptake indicate that increasing CSC replacement enhanced the extent of carbonation and mineral CO_2_ sequestration under the investigated conditions.
- CSC50 combined a 50% reduction in OPC consumption with a net CO_2_ uptake of 50.5 kg per ton of concrete, demonstrating the potential to combine OPC substitution with direct CO_2_ sequestration. However, this uptake does not represent the net reduction in greenhouse gas emissions over the production process. A comprehensive life-cycle assessment incorporating raw-material production, CSC calcination, steam curing, CO_2_ supply, and carbonation-curing energy is required to establish the actual carbon-reduction potential.

The findings support further development of carbonation-cured CSC-containing concrete, while its structural applicability requires additional verification. Future studies should evaluate elastic modulus, tensile and flexural strengths, long-term deformation, and durability, together with the effects of member dimensions and curing conditions beyond the laboratory environment using 20% CO_2_. For reinforced applications, depth-dependent alkalinity, reinforcement corrosion, bond performance, and cracking behavior should be assessed to address the potential consequences of carbonation-induced alkalinity loss. The compatibility of GFRP reinforcement with carbonation-cured CSC-containing concrete should also be investigated.

## Figures and Tables

**Table 1:** Chemical compositions of OPC and CSC.

Materials	Oxide Contents (wt%)										
	CaO	SiO_2_	Al_2_O_3_	Fe_2_O_3_	SO_3_	MgO	TiO_2_	Na_2_O	K_2_O	MnO	P_2_O_5_
OPC	61.2	20.7	5.2	2.9	2.6	2.9	0.3	0.3	0.9	0.1	0.2
CSC	41.5	53.2	2.1	0.7	-	1.3	0.1	0.2	0.6	-	-

**Table 2:** Physical properties of aggregates.

Materials	Apparent Density (kg/m^3^)	Fineness Modulus
S	2.61	3.14
G	2.59	7.02

**Table 3:** Mix proportions of CSC concrete.

Mixture	W/B (%)	S/a (%)	Unit Weight (kg/m^3^)					SP (B×%)	AE (B×%)
			W	OPC	CSC	S	G		
CSC0	45.5	48.6	167	367	0	763	907	0.64	0.064
CSC25	45.5	48.6	167	275	92	759	902	0.72	0.072
CSC50	45.5	48.6	167	183.5	183.5	755	897	0.67	0.067

**Table 4:** Rietveld-derived quantitative phase compositions of paste specimens.

	CSC0			CSC25			CSC50		
	Ctrl	3 D	14 D	Ctrl	3 D	14 D	Ctrl	3 D	14 D
C_3_S	4.7 ± 0.1	2.9 ± 0.1	1.3 ± 0.2	1.6 ± 0.1	1.0 ± 0.3	0.6 ± 0.1	1.2 ± 0.1	0.5 ± 0	0.3 ± 0.2
C_2_S	13.4 ± 0.4	9.5 ± 0.3	7.2 ± 0.1	16.0 ± 0.5	8.2 ± 0.6	5.3 ± 1.7	15.5 ± 0.3	3.9 ± 0	3.0 ± 0.1
C_3_A	0 ± 0	0 ± 0	0 ± 0	0 ± 0	0 ± 0	0 ± 0	0 ± 0	0 ± 0	0 ± 0
C_4_AF	0.6 ± 0.1	0.3 ± 0.1	0.4 ± 0.1	0.3 ± 0.2	0 ± 0	0.1 ± 0.1	0 ± 0	0.2 ± 0.1	0.3 ± 0.1
C_3_S_2_	0 ± 0	0 ± 0	0 ± 0	2.8 ± 0.7	1.4 ± 0.4	1.9 ± 0.6	5.6 ± 0.7	1.5 ± 0.4	1.1 ± 0.3
CS	0 ± 0	0 ± 0	0 ± 0	7.6 ± 0.4	6.6 ± 0.4	7.3 ± 0.7	15.0 ± 0.7	8.3 ± 0.5	6.0 ± 3.1
Ca(OH)_2_	14.4 ± 0.1	9.0 ± 0.2	2.4 ± 0.1	12.4 ± 0.2	2.4 ± 1.4	0.9 ± 0.1	8.2 ± 0.3	0.5 ± 0.2	0.3 ± 0.2
Cal	4.5 ± 0.7	21.3 ± 0.5	42.6 ± 0.1	5.1 ± 0.4	34.2 ± 4.1	41.9 ± 0.8	3.3 ± 0.3	29.5 ± 0.5	37.2 ± 0.3
Vat	0.1 ± 0.1	0.4 ± 0.3	1.9 ± 0.2	0.3 ± 0.1	5.5 ± 0.4	4.5 ± 0.2	0.1 ± 0.1	10.4 ± 2.1	15.1 ± 0.7
Ara	0.3 ± 0.2	0.2 ± 0.3	1.1 ± 0.4	0.4 ± 0.1	1.1 ± 1.5	0.6 ± 0.3	0 ± 0	0.8 ± 0.1	2.9 ± 0.1
AFt	0.5 ± 0.2	0.5 ± 0.2	0.2 ± 0.3	0.4 ± 0.5	0.2 ± 0.2	0.1 ± 0.1	0 ± 0	0 ± 0	0 ± 0
Cris	0 ± 0	0 ± 0	0 ± 0	1.9 ± 0.1	1.7 ± 0.3	1.0 ± 0	3.8 ± 0	3.0 ± 0.2	1.8 ± 0.1
ACn	61.4 ± 1.8	56.2 ± 1.9	43.1 ± 1.4	51.3 ± 3.4	37.8 ± 1.9	36.0 ± 1.1	47.5 ± 1.9	41.6 ± 4.0	32.3 ± 3.1

Cal: Calcite; Vat: Vaterite; Ara: Aragonite; AFt: Ettringite; Cris: Cristobalite; ACn: Amorphous and crystalline non-quantified.

**Table 5:** Mass loss and net CO_2_ uptake of concrete specimens determined by TGA (500–800 °C).

Mixture	Before Carbonation Curing (wt.%)	After 14 Days of Carbonation Curing (wt.%)	Net Mass Loss (wt.%)	Net CO_2_ Uptake (kg-CO_2_/t-Concrete)
CSC0	0.87	2.69	1.82	18.2
CSC25	0.88	4.28	3.40	34.0
CSC50	0.83	5.88	5.05	50.5

## Data Availability

The original contributions presented in this study are included in the article. Further inquiries can be directed to the corresponding authors.

## Abbreviations

OPC	Ordinary Portland cement
CSC	Low-lime calcium silicate cement
C_3_S	Tricalcium silicate (Alite)
C_2_S	Dicalcium silicate (Belite)
C_3_A	Tricalcium aluminate
C_4_AF	Brownmillerite
CS	Pseudowollastonite
C_3_S_2_	Rankinite
XRF	X-ray fluorescence spectroscopy
XRD	X-ray diffraction
TGA	Thermogravimetric analysis
FT-IR	Fourier Transform Infrared
